# Neurobehavioral Phenotype and Dysexecutive Syndrome of Preterm Children: Comorbidity or Trigger? An Update

**DOI:** 10.3390/children9020239

**Published:** 2022-02-11

**Authors:** Catherine Gire, Aurélie Garbi, Meriem Zahed, Any Beltran Anzola, Barthélémy Tosello, Valérie Datin-Dorrière

**Affiliations:** 1 Department of Neonatology, North Hospital, University Hospital of Marseille, Chemin des Bourrelys, CEDEX 20, 13915 Marseille, France; catherine.gire@ap-hm.fr (C.G.); aurelie.garbi@ap-hm.fr (A.G.); meriem.zahed@ap-hm.fr (M.Z.); any-alejandra.beltran-anzola@ap-hm.fr (A.B.A.); 2 CEReSS—Health Service Research and Quality of Life Center, Faculty of Medicine, Aix-Marseille University, 27 Boulevard Jean Moulin, 13005 Marseille, France; 3 CNRS, EFS, ADES, Aix Marseille Universite, 13915 Marseille, France; 4 Department of Neonatal Medicine, Caen University Hospital, Avenue Cote De Nacre, 14000 Caen, France; datindorriere-v@chu-caen.fr

**Keywords:** extremely pre-term children, neurocognitive/behavioral disorders, executive function, neurodevelopment

## Abstract

Premature birth is a worldwide public health priority. One in ten children is born before 37 weeks of gestational age and, in developed countries, survival rates without major neonatal morbidity are increasing. Although severe sequelae associated with these births have decreased, their neurobehavioral difficulties, often associated in multiple fields, remain stable but still widespread. These neurobehavioral difficulties hamper the normal development of academic achievements and societal integration and intensify the children’s needs for rehabilitation during their preschool and academic years. Severe sequelae increase when gestational age decreases. This is even truer if the socio-cultural background is impeded by low income, education and language skills as compared with defined averages. However, moderate and/or minor neurocognitive and/or behavioral difficulties are almost identical for a moderate or a late preterm birth. Obtaining a better clinical description of neurobehavioral characteristics of those pretermly born, once they reach preschool age, is essential to detect behavioral issues as well as early specific cognitive difficulties (working memory, planning, inhibition, language expression and reception, attention and fine motor skills, etc.). Such information would provide a better understanding of the executive functions’ role in brain connectivity, neurodevelopment and neuroanatomical correlation with premature encephalopathy.

## 1. Introduction

Comprehensive research over the past three decades has profiled the neurodevelopmental consequences of preterm birth. Nonetheless, relative to our understanding of the motor, cognitive and behavior outcomes, robust conclusions regarding the true nature of neurobehavioral outcomes of premature children remain a major challenge, spanning from subclinical to clinical presentations. The objective of this review is to provide a complete and current characterization of the neurobehavioral phenotype and highlight the main gaps in knowledge, mainly with regard to the evolution of symptoms, the co-occurrence of disorders in the same individual, associations with chronological age and degree of prematurity. Hypotheses suggest that this neurobehavioral phenotype of prematurity is due to brain hypo-connectivity secondary to encephalopathy of prematurity. Currently, one long-term study describes a true “typical” neurobehavioral profile of the premature child with all the different aspects of development, namely, cognition, attention, executive function, motor skills and behavior [1].

In line with the Preferred Reporting Items for Systematic Reviews and Meta-Analyses (PRISMA) guidelines, a search was undertaken on PubMed/MEDLINE and the first 10 pages of Google Scholar electronic databases for peer-reviewed, original publications between 1 January 2011 and 31 December 2021, using the primary search terms “premature neurobehavioral phenotype” and “premature behavioral phenotype and dysexecutive syndrome” were considered. To ensure comprehensiveness, a supplementary search was performed with the search terms “premature” AND “phenotype” OR “premature phenotype” AND “executive function” OR premature phenotype and disability OR Premature and “psychiatric” OR “attention-deficit/hyperactivity disorder” OR “inattention” OR “autism spectrum disorder” OR “social” OR “social–emotional” OR “anxiety” OR “internalizing”/“internalising” or dysexecutive syndrome, or minimal motor disorder. Studies were considered for inclusion in this review if they investigated the neurobehavioral and/or emotional outcomes and/or dysexecutive syndrome and/or neurodisabilities of children and adolescents born premature, and if they made explicit reference to the premature neurobehavioral phenotype in the background or discussion of the study when interpreting the findings. The objective of this narrative review is to describe, as precisely as possible, the long-term neurobehavioral profile of the premature infant, its characteristics compared with those of the infant born at term and the relevance of this assessment, that is, the social consequences and/or quality of life and the role of executive functions in the genesis of the profile. Thus, we approach the neurobehavioral sequelae of prematurity according to different perspectives, by sequelae and by neurobehavioral disorders.

## 2. Prematurity, Mortality, Morbidity and Long-Term Sequelae: The Classical Description of the Long-Term Outcome

### 2.1. Mortality

Regarding the mortality of children under 5 years old, prematurity is the leading cause of death in this age category for both developing countries (from 10 to 20% of deaths before the age of 5 years) and developed countries (23%) [2]. The overall survival rate of VPs (<32 weeks) has increased in many countries over the course of the past decade [2,3,4,5,6,7]. In the case of extreme prematurity, the results are more mixed (EPICure cohort 1 and EPICure cohort 2 [4]; EPIPAGE 1 cohort and EPIPAGE 2 cohort [5]). The heterogeneity of definitions makes comparisons between studies difficult [4,8]. In a recent, large international cohort of 11 countries focused on VP-born children, mortality declined between 2007 and 2015 and, except for Canada, there was an increase in bronchopulmonary dysplasia in most countries [3,9].

### 2.2. Cerebral Palsy and Gross Motor Disabilities

The frequency and severity of cerebral palsy (CP) have decreased in recent years due to advances in perinatal medicine, such as corticosteroid therapy, magnesium sulfate, developmental care and nutritional techniques [8]. The drop in the incidence of CP at two years of corrected age from 17% in 1997 (EPIPAGE 1) to 8% in 2011 (EPIPAGE 2) for children born at 25–26 weeks of gestational age (GA) [9] was confirmed at the European level [5,10,11,12]. EPIPAGE 2 or EXPRESS [13,14] studies have shown a severe cognitive deficit at the age of 5–6 years, in 10–15% of extreme GA groups (<27 weeks of GA) [15,16]. In the recent meta-analysis by Twilhaar (71 studies, 7752 VPs), 16% of VPs had an FSIQ <−2DS, versus 2.5% in term-born children [13].

### 2.3. Cognition

- Global cognitive deficits

If mortality and so-called severe morbidity have generally decreased, preterm birth, even moderate, remains a risk context for neurodevelopmental sequelae and educational difficulties, which are more frequent with a low birth term and when the child’s environment is socio-economically disadvantaged [8]. Various recent studies on VP children have shown a clinically significant difference of 13–15 FSIQ points, or from −0.85 to −1 SD, compared to term children [13,14,15,16]. A moderate cognitive deficit (FSIQ between −1 and 2 SD) was found in a little more than 25% of VP babies when they reached five years of age. This appeared to be true even in children born moderately premature (32–34 weeks of GA) vs. 10% in the control group children born at term in the EPIPAGE 2 study [11]. There are many methodological biases to consider in describing medium- and long-term cognitive outcomes [11,17,18]. Taking the parents’ educational level and socio-economic status into account is essential in any cognitive assessment [19,20]. The “classic physiological” neurodevelopment is modified by preterm birth and the neurobehavioral sequelae found in preterm children do not differ radically from the “dys” problems encountered in term births. Their specificities are greater incidence and complexity; intricacy with behavioral disorders and/or coordination acquisition disorders (CADs); and characteristic prevalence of dysexecutive and attentional disorders [21].

- Language skills

Language is one of the most affected functions in the preterm infant [22,23]. Language skills were found to be poorer in preterm children than in full-term infants, with a performance of approximately from −0.5 to −1.0 SD in each language domain studied [23]. This finding was supported by functional MRI which confirmed a language circuit dysfunction in premature children during language processing with a predominance of expressive delay [24].

- Fine motor skills and coordination acquisition disorders

Dyspraxia (or CAD, in the international nomenclature) is a disorder in the development of gestural functions in a context of more or less marked deficit of spatial treatments. In VPs, CAD is currently much more frequent than CP. It is present in 18.8% of the VPs born at 24–26 weeks of GA, in 8.5% of the VPs born at 27–31 weeks of GA and in 5% of the VPs born at 32–34 weeks of GA compared with the control population at term, as reported in the EPIPAGE 2 study [11,25]. Furthermore, the VP-born infant has significant dysfunctions in a range of basic cognitive processes, such as working memory (WM), processing speed, visuo-perceptual skills, sensorimotor integration and attention, as compared with term-born infants [26]. Such deficits, due to the impairment of executive functions (EFs), are observed from when they enter school until the time they reach adolescence [27,28]. Several meta-analyses have shown differences in inhibition, WM and planning varying from 0.3 SD to 0.6 SD in premature children as compared with children born at term. This difference is stronger at the youngest GA and worsens over time for WM [15,18,29,30]. Risk factors for VPs’ executive deficits can be summarized into four categories, namely, immaturity (weeks of GA), growth restriction, perinatal inflammation/infection and socioeconomic disadvantages [31]. EF deficits are also reported by parents and teachers in preterm infants and appear to persist over time [32,33,34]. Preterm infants at the age of 5 years have shown poorer performance in visual attention than children in full-term control groups, with no differences in other cognitive abilities [35,36]. These visuomotor integration problems persist into childhood and adolescence [37,38].

- Behavioral and psychiatric disorders

Preterm birth is associated with a psychopathological risk that can occur in isolation or can be associated with neurocognitive disorders and/or learning disabilities [21]. Although results vary considerably among the rating scales completed by the parents or teachers, the prevalence of psychiatric disorders is three times higher in preterm children than in term-born control groups [39]. A number of studies concerning mid-childhood and adolescence describes a “premature behavioral” phenotype characterized by inattention, anxiety and social difficulties [40]. Three types of behavioral phenotypes are described in the psychiatric disorders observed in VP cohorts, (1) ADHD (symptoms of inattention rather than hyperactivity/impulsivity); (2) emotional disorders (anxiety rather than depression); and (3) ASD, autism spectrum disorder, social interaction and communication problems [27]. Additionally, VP preterm infants have poorer peer relationships and weaker social competence, without major abnormalities in other areas [41]. Elevated autism-spectrum-disorder rates, particularly disorders of facial emotion recognition, have been noted to vary from 5 to 8% in adolescence or adulthood [42]. Recently, in EPIPAGE 2, among 5.5-year-old VP-born children, subgroups could be distinguished with distinct outcome profiles that varied in severity, type and combinations of deficits, with a worsening of neurocomorbidities associated with behavioral disorders [43]. The behavioral disorder characteristic of the child or adolescent born pretermly is the absence of conduct disorders [44]. Academic repercussion for the pretermly birth has a strong economic impact, since lower educational levels in adulthood yield more unemployment, under-qualified work, anxiety disorders and loss of self-esteem [45]. Academic failures increase with the decrease in the child’s gestational age. However, as reported in a recent review, moderately preterm children are also at risk of learning disabilities in reading and mathematics [46]. In Twilhaar et al. [13], 2390 children showed performance scores of −0.71 SD in mathematics, −0.44 SD in reading and −0.52 SD in spelling (confirmed by Allotey, with scores of, respectively, −0.78 SD, −0.67 SD and −0.56 SD) in VP children aged from 5 to 8 years as compared with the control group born at term [14]. A meta-analysis of 33 studies covering 4000 premature infants confirmed this hiatus in school performance (math and reading) between VP children and their peers [47]. In the recent EPIPAGE 2 study, around half of the children born between 24 and 26 weeks of GA received at least one paramedical care or school support service. This decreased to 26% for children born between 32 and 34 weeks of GA [11].

The difference in academic results persists in secondary educational levels in adolescence and adulthood, indicating a lesser chance to integrate into high school or university [14,48,49]. This association was observed to be greater when the FSIQ was <85 (−1 SD). From the perspective of both children and parents, the quality of life (QOL) of school-aged VP children with no serious impairments was lower than that of a reference population [50]. Language delays, visual perceptions, dysexecutive and behavioral disorders are determinants of this QOL [51]. Neurobehavioral disorders continue in those VP adults with no severe impairments. These adults have a less-than-optimal QOL compared with those adults born at term. Their QOL continues to worsen from adolescence into adulthood [52].

The international and temporal consistency of the results on the neurodevelopmental outcome of VP babies confirms that the disruption of biological (pro-inflammatory factors, growth factor deprivation, etc.), anatomical (disruption of the architecture and establishment of brain connectivity) and environmental factors (transition from a uterine environment to a dys-stimulating neonatal intensive care environment) underlies neurodevelopment and, subsequently, leads to a diffuse and protean clinical impairment [26]. With the decrease in severe sequelae, neurodevelopmental disorders of long-term prematurity become predominant. They present as a “diffuse neurobehavioral” disorder with an impairment of multiple “functions”, such as cognitive, predominantly executive and/or motor and/or behavioral functions, and have a significant impact on social adaptation and/or quality of life [50,51]. It is, therefore, necessary to have a global approach to the neurodevelopmental sequelae of prematurity by considering all the functional alterations and, above all, behavior and/or EFs. Finally, a holistic view of neurodevelopment is more suited to the reality of the assessment of clinical sequelae of prematurity [8]. Sequelae are observed in several domains (multi-dys) due to their close interactions during development, with a predominance of dysexecutive syndrome, testifying to the importance of sensorimotor interactions for the development of learning and behavior. It is under this new perspective that the neurodevelopmental outcome of five-year-old children in the EPIPAGE 2 cohort was recently presented with a composite score of evaluation of “neurobehavioral” disorders at four levels, namely, no deficit, mild deficit, moderate deficit, or severe deficit, including the analysis of the intelligence quotient (Wechsler IV); the screening of visual and auditory disorders; the study of motor disorders (cerebral palsy (CP), via the Gross Motor Function Classification System (GMFCS), and coordination acquisition disorder (CAD) via the Movement Assessment Battery for Children—Second Edition (M-ABC2)); and behavior (Strengths and Difficulties Questionnaire (SDQ)). With this new classification, more than one-third of VPs were found to have mild neurodevelopmental morbidity, including moderate premature infants (32–34 weeks of GA). This approach makes it possible to better evaluate the neurodevelopment of the premature infant in its quasi-globality, since the alteration in EFs is not taken into account in a direct way. However, neurobehavioral disorders correspond more to the reality of the current follow-up, where mild-to-moderate deficiencies have a significant impact on learning and on the quality of life (QOL) of these children [11].

## 3. Understanding the Neurodevelopment of Prematurity Requires Executive Function Evaluations

EFs define the cognitive operations that allow the individual to adapt their behavior and activities to the demands and fluctuations of the environment. These functions come into play as soon as the individual is faced with a non-routine situation that requires problem solving.

EFs’ main mental processes are: (1) planning, i.e., organizing and planning data according to the goal to be achieved and choosing relevant information; (2) inhibition, i.e., inhibiting secondary processes, resisting distractions; (3) working memory (WM), i.e., organizing for memory reuse; (4) flexibility, i.e., implementing treatment operations, inventing new situations and being able to modify them if they deviate from the desired goal [53,54]. It is understood that each of these mental processes (“under executive mental functions”) can be evaluated by “specific” tests, but these processes are often entangled and dependent on the attentional mental process (auditory and/or visual). For this reason, their definitions vary significantly from publication to publication [54,55]. FEs are, therefore, “higher” functions that play an important role in cognitive neurodevelopment and social adaptation [56]. The EFs are a set of high-level cognitive processes that guide our actions, regulate our behavior and allow us to adapt to our environment to achieve a specific goal.

Cohort descriptions of a premature infant’s fate are usually classified according to the severity of the disability based on the full-scale intelligence quotient (FSIQ) score, namely, no disability, mild disability, moderate disability, or severe disability. However, a low intelligence quotient (FSIQ) is the product of social disadvantage, genetic influences, great prematurity and other environmental factors that play a greater role over time. VPs require more discriminative neuropsychological and behavioral analyses to identify all the affected functions [26]. Indeed, an FSIQ threshold above or close to a low mean (Wechsler) is generally considered “normal” in cohorts of premature infants. However, the performance observed is the result of complex processes involving multiple intellectual and non-intellectual characteristics, such as attention, emotions, motivation, movement planning, EFs, etc., which may be in deficit in premature infants. Thus, the FSIQ calculated in premature infants on subtest values, which are most often dissociated, does not reflect the child’s cognitive functioning. Rather, it is the analysis of the Wechsler subtest dispersion that should highlight those children that deserve a thorough interpretation, therefore a better brain function assessment. A child born prematurely and viewed with a “normal” FSIQ may also present a dysexecutive syndrome and/or an alteration in behavior that may indirectly disrupt his/her cerebral functioning [57]. Without taking into account any behavioral analysis, the study by Heeren [57], on the Elgan cohort, described four types of neurocognitive profiles, one being normal and two profiles (moderate and severe profiles) presenting diffuse cognitive and executive function impairments, while the impairment is mainly dysexecutive for the fourth “normal low profile” group. Heeren’s study demonstrated that FSIQ measurements were insufficient to characterize moderate or minor cognitive impairment resultant of extreme prematurity because the impact of EF disorders, such as inhibition, WM and mental flexibility, was minimized. A recent study showed that a cluster analysis taking into account the whole cognitive development (including EFs) and a behavior analysis defined three distinct outcome groups in extreme premature children without severe disabilities, providing an informative means for identifying factors related to developmental outcomes. This study showed that QOL deterioration was determined by the severity of the three neurobehavioral “phenotypes” and was also defined by dysexecutive and/or behavioral disorders [1].

### 3.1. Executive Functions and Learning in Premature Infants

EFs oversee lower-level cognitive processes, hence the term “top-down”; thus, they are at the center of overall cognitive functioning, such as a true “orchestra conductor”; they notably play a preponderant role in academic achievements [56,58,59,60,61,62]. The better EFs are in young children, the better are their results in mathematics [55,63,64,65,66]. Similarly, a higher level of reading comprehension in young children is associated with elevated EFs [67,68]. A timely assessment of EFs, from early childhood, is predictive of school performance [69,70,71]. EFs (especially WM) are more predictive of academic success than FSIQs [72,73] and reflect the child’s degree of educational investment [74]. EFs are not only involved in cognitive and learning mechanisms, but also in the regulation of behavior and of emotions. A direct link has been suggested between EFs and QOL in children [50,75]. This EF centrality to cognitive development not only has a persistent impact on adulthood and career success [76] but also on behavior and emotional well-being [77]. Thus, EFs are involved in all areas of our lives, at all ages [78,79] and the particular impairment in premature children must be emphasized. Tatsuoka showed that counting difficulties beginning in kindergarten for VPs were linked to an executive deficit, unlike full-term children who might experience mathematical difficulties [80]. Loe showed a link between parent-reported Behavior Rating Inventory of EFs (BRIEF) and reading difficulties in premature children between 9 and 16 years of age [81]. Aarnoudse-Moens, in 2013, in a study on a series of 200 children born <30 weeks of GA (median age of 8 years), concluded that the executive deficit, beyond the impact of FSIQ, had an impact on mathematical skills and attentional difficulties compared with children born at term [82]. Dai et al. [83] underlined the links between FSIQ (WISC-IV), BRIEF, Test of Everyday Attention for Children (TEA-Ch) and school learning in a cohort of 70 children born <30 weeks of GA. These children were assessed at the age of 7 years old with the conclusion that FSIQ impacted school learning, mainly through EFs. Twilhaar, in a 2020 study on 13-year-old VP-born adolescents, reported that the role of the executive deficit (WM deficit, processing speed and attentional processes) influenced the difficulties in learning mathematics and reading comprehension. She suggested that the screening of these executive deficits enabled the targeting of children who were at risk of these educational difficulties [84].

Mulder demonstrated, in fifty ten-year-old preterm births (<31 weeks of GA), that processing speed and WM were preponderant factors for the acquisition of various academic skills (liberal arts and math). He concluded that a 10 min rapid assessment of WM and processing speed could provide a rapid screening of children requiring specific learning support [85,86]. Furthermore, the inhibitory skills of 18-month-old VP infants could predict attention and learning difficulties by the age of 8 [87]. A deficit in inhibitory control to correctly process visual information in VP children has important academic implications for VPs, since it has been shown, for example, that performance in visual search tasks is correlated with reading skills [88]. Intermediate and overall visual processing difficulties may partly explain reading difficulties, among other academic impairments, reported in VP children [84,89,90]. We also highlight the higher cognitive cost for VP children, which consists of inhibiting visual distractors present in their environment.

### 3.2. Executive Functions, Behavior and Attention Disorders

A meta-analysis of behavioral profiles of school-aged and adolescent VP children, as compared with those children born at term (2004 premature versus 1238 controls), showed that behavioral profiles, according to their severity, are specific and associated with cognitive and/or neurological comorbidities [91,92]. The authors proposed, for the first time, the concept of “behavioral phenotype of premature infants”, characterized by attention deficit/hyperactivity disorder, social and emotional difficulties and introversion [21,91,92,93]. Burnett confirmed this association with worsening behavioral disorders when associated with neurological comorbidities [27] (Table 1). A cluster analysis identified four behavioral profiles in five-year-old VP children, i.e., (1) children with a typical development similar to that of the general population; (2) children at “risk”, with neurodevelopmental scores and psychiatric profiles slightly disturbed, but close to the mean; (3) children with moderately severe to severe executive disorders and symptoms of ADHD and/or ASD; and (4) children in inattentive/hyperactive groups with cognitive and linguistic scores close to the deficit [94].

In 2019, Burnett [98] reported that VP children, aged from 8 to 12 years, with high behavior disorders were associated with parental education levels, FSIQ, visuospatial WM and lower inhibition. This suggests that impaired executive functioning plays an important role in all neurobehavioral mechanisms of prematurity [99,100]. Fitzallen et al. described specific behavioral phenotypes of VP children with attention disorder, autism spectrum disorders and anxiety disorders [21]. Links between this behavioral phenotype of the child born prematurely (20%) and educational outcomes have been highlighted [98], underpinned by executive mechanisms. Twilhaar reported that VP adolescents who had difficulties in social relations showed a significant correlation with executive deficit [95,101]. Finally, it was shown that former VP children with an FSIQ greater than 85 had social adjustment disorders in 16% of cases associated with attention deficit and executive, language, communication and emotional disturbances [79].

Beginning with kindergarten, VP-born children need more attention from the teacher because they are less focused on the tasks at hand [102]. These preschool attention disorders are associated with behavioral and learning difficulties [103] and the impact on the early development of social relationships persists over time [104]. Behavioral studies of premature infants have reported they have more attention deficit disorders and poorer socio-emotional competence [105]. Studies of three SDQ parental questionnaires have shown a significant incidence of problems concerning emotional behavior, inattention, hyperactivity and peer relationships [106,107,108]. These disorders were significantly less frequent when the mother had received antenatal magnesium sulfate [109] and had an inverse relationship with GA at birth.

Attention is a complex process involving several cognitive processes of visual attention, orientation, alertness and executive attention, such as WM. The deficits observed in EFs may explain the attention problems observed in extreme preterm births [110,111]. In the preterm child, attentional deficits are linked to a dysexecutive syndrome, such as a visuospatial WM and/or inhibition disorder [89]. After adjustment for FSIQ, visuospatial scores were lower in school-age VP children than in full-term children [112]. An association between attentional disorders, low processing speed and WM has been frequently found [86,89]. Lower gestational age and cognitive deficits are correlated with lower visuomotor performance in premature infants regardless of birth weight, age at testing and year of birth. Atkinson (2017) reported significant deficits in visual attention and in many visual cognitive tasks in VP children aged from 6 to 7 years, despite their relatively normal language tests and FSIQ [113]. It has been shown that visuospatial impairments in premature infants also concern gross motor integration [114,115]. A cohort study of VP school-aged children without serious sequelae showed that CAD were most often associated with comorbidities such as behavioral and/or executive disorders and/or attention disorders [96]. In contrast, behavioral disturbances can be present identically in premature infants with or without CAD. A diagnosis of CAD, therefore, justifies a behavioral measurement and/or an EF/attention measurement.

The analysis of a visual scene comprises different hierarchical levels of information, from the most local to the most global elements. All local information can be individualized or integrated into a more comprehensive structure, which can be modelled in the form of hierarchical figures [116]. In healthy children, the strategy of global/local visual processing evolves with age; the youngest children present a phenomenon of local precedence (local visual bias), while those over 6 years old have an adult-type preference for global visual information [117,118,119]. This global precedence effect is characterized by a faster and more efficient global processing than the local processing and an interference effect of the global information during the local processing. The global precedence effect relies on the fact that attention resources are primarily allocated to the global structure, which is processed more easily and efficiently than local elements [120]. When global and local information conflict, the inhibitory control, an EF, plays a crucial role in overcoming efficient and automatic global processing in order to focus attention on local information processing, starting in childhood [120,121]. Several global/local visual attention studies found atypical performances in 6–9-year-old VP children who appeared to use a local visual strategy when processing global configuration stimuli which resulted in significantly lower scores than the control groups [122,123]. Similarly, the Helsinki study on 25-year-old adults born with very low birth weights (VLBW < 1500 g) explored the visual processing during the Rey–Osterrieth figure test and Block Design of WISC-III; a slower overall visual processing speed was observed, but their local visual processing was not affected in adults born with VLBW compared with adults born at term [124]. VP infants are particularly at risk of developing global/local visual processing difficulties and this appears to be, at least in part, mediated by executive deficits [125]. Recently, amongst 22 VP-born 10-year-old children and 21 term-born children, Dorrière [125] showed the following: (1) preservation of local visual processing, even with complex stimuli (three hierarchical levels of visual information), a maturing deficit in visual attention, with a phenomenon of local precedence (as in the younger child) rather than a phenomenon of global precedence, as observed in children of the same age who were term-born; (2) a deficit in inhibitory control with a significant slowing of processing speed in the presence of a number of visual distractors (three and more) compared to term children. Compared with dyspraxia child born at term, dyspraxia premature infant performed significantly more poorly in visual attention and sensorimotor precision, while their visuomotor skills were similar [37].

### 3.3. Executive Functions and Holistic Neurodevelopment

Hutchinson [126] and Anderson [26] hypothesized the impairment of original primary cognitive functions. For example, a WM deficit and/or attention and/or processing speed impacting other mental processes would be the cause of later deficits such as language delays or dysexecutive disorders [85,86]. A lexical stock study of prematurely born infants in the EPIPAGE 2 cohort showed a strong association between language skills and performance in other areas of development when they reached a corrected age of 24 months and confirmed that the neurodevelopment of the premature infant should be considered with a holistic approach during infancy [127]. The child’s language implicitly develops on pre-linguistic sensorimotor skills. Sensorimotor constraints affecting oral and facial praxis, auditory and tactile discrimination, visual attention and modality transfers are observed in premature children with phonological disorders [128]. A cluster-based fate analysis approach illustrated the holistic neurodevelopment of VLBW infants from birth to 18 months. The majority of children were cognitively normal, but gathered into three different groups, that is, (1) 17% with cognitive and language results above the standards of the tests used; the majority of children (54%) in the middle range for cognition, expression and reception of language; (2) 21% with an average score for cognition and language reception, but with a notable delay in language expression; and (3) 8.5% with poor performance in all areas of cognitive and language development. This classification made it possible to raise the hypothesis of an attentional problem at the origin of these disorders and to consider surveillance for all groups except for those in Group 1. In the event of a disadvantaged socio-economic environment, reinforced surveillance, when they achieve school age, is essential [97]. Delays in the development of oral language are common in both expression and reception in VP infants. These language difficulties seem to increase as the language becomes more complex, i.e., from age 3 to 12. Language plays a special role in learning abilities and its achievement is based on intellectual functions allowing the child to achieve non-verbal communication and requiring a high level of sensoriality, perception, attention and fine motor skills. It is a necessary function for the construction of cognitive development and social relationships [127]. This result was confirmed by a recent study in EPIPAGE 2 showing that, among 5.5-year-old very premature born children, subgroups could be distinguished with distinct outcome profiles that varied in severity, type and combinations of deficits [43].

### 3.4. Neurobehavior, Executive Functions and Hypoconnectivity

Executive functions are localized in the fronto-striatal and fronto-parietal circuits. They are affected by ADHD and ASD and by very preterm births [129]. The neurobehavioral disorder of prematurity is linked to a lesion and/or environmental mechanism affecting the development of the brain and corresponding to common neuroanatomical lesions [130,131,132,133].

Following the analysis of 100 MRI scans, Inder described the general “pattern” of non-cystic brain lesions of VP infants with both white matter and grey matter (GM) abnormalities, which included white matter atrophy, ventriculomegaly, delayed gyration and enlargement of the brain spaces under the arachnoid. The perinatal risk factors identified for these lesions included weeks of GA, infectious episodes, neonatal hemodynamic disorders and cerebral ultrasound lesions (Hemorragia intra ventricular (HIV) and periventricular leucomalacia [134]. Volpe emphasized that the observed lesions of white matter in VP infants were associated with diffuse neuronal and axonal abnormalities of the white matter but also of the cortical grey matter, thalami and basal ganglia, as well as the cerebellum. He suggested the term “premature encephalopathy”, witness to initial lesions and secondary developmental alterations leading to dysmature evolution of the brain of a child born prematurely [132,133].

MRI scans performed later in childhood, or even adolescence, on VP children and the application of different anatomical and functional MRI techniques enable better descriptions of these anomalies. For example, the DTI (diffusion tensor imaging) analysis, an anatomical diffusion MRI, defines more clearly these anatomical white matter lesions since the areas with signal abnormalities also show changes in the ADC (apparent diffusion coefficient), anisotropy fraction (AF) and radial diffusivity, indicative of altered oligodendroglia [135,136,137,138]. Direct links among these brain abnormalities observed via MRI performed at term and neurobehavioral difficulties have been demonstrated in premature infants, e.g., white matter and global neurodevelopment (Bayley scale) at 18 months of corrected age [139]; white matter and cognitive abnormalities between the ages of 4 and 6 years (as compared to a term birth control groups) [140]; association between language disorders in childhood (aged 7 years) and qualitative abnormalities of the white matter [141,142]; and link between diffuse white matter abnormalities (reduction in AF, anisotropy fraction, to DTI) and attentional and anxiety disorders, thus witnessing an abnormality in global connectivity [81,143]. In school-aged VP children, a reduction in FA in DTI was found in the putamen and insula regions correlated with defect in WM and mathematical skills and in white matter in cortical regions correlated with inhibitory control (IC) [142,144]. Other studies have focused on WM and its association with qualitative white matter abnormalities [145] or volume anatomical abnormalities, such as reduction in hippocampal volume [146]. In addition, links between MRI spectrographic abnormalities and executive deficit in VPs at age 8–13-years have been demonstrated [147]. Finally, other studies in VP adolescents or adults have reported these links between executive deficit and brain abnormalities using MRI [148,149].

## 4. Perspectives, Executive Functions and Analysis of Neurobehavior

To date, various long-term follow-up studies have almost exclusively examined the gross aspects of development, such as cognition and/or language. The examination of EFs and/or of behavior and/or of fine sensorimotor areas is poorly reported or is reported with comorbidities [97].

As well as being a health issue [8,150,151], a better understanding of the long-term future of children born prematurely under all these aspects is essential, since it provides parents with scientifically based and reliable information and improves care thanks to the understanding of neurodevelopmental disorders [1].

Using data from recent meta-analyses, international cohort comparisons indicate that rates of survival and rates of neurodevelopmental disorders in premature infants show that many methodological issues must be taken into consideration in describing long-term outcomes. In fact, the description of neurodevelopmental disorders depends on the population analyzed, the method of assessing, gestational age, the year of birth, the intensity of perinatal care and postnatal care, the age of neurobehavioral assessment, the follow-up rate, types of tests and measures used and the analysis of results [17].

In order to draw solid conclusions about the specific neurobehavioral profiles that exist in the general VP population, the identification of clustered subtypes needs to be performed in large cohort studies with measures of brain cognitive, motor, executive and behavioral functioning [1,43]. Future studies should also study the trajectories of different neurobehavioral profiles in adulthood, to examine whether neurobehavioral clusters are stable over time so as to better understand their future management prospects.

## 5. Support for the Development of EFs and Neurobehavioral Functions in Children Born Premature

Apart from those who present serious sequelae specific to their type of CP or cognitive impairment, the neurodevelopmental outcome of the VP infant is characterized by a set of minor-to-moderate dysfunctions in the developmental fields (language, praxis, executive, behavioral and attention disorders, social interaction disorders, etc.). These dysfunctions tend to cumulate, even to potentiate, which impacts school learning and the daily life of these children and their parents [13,14,15,45,51,100,101,152]. Executive functions, such as high-level cognitive operations, play a preponderant role in learning and social adaptation via the regulation of children’s behavior and emotions [56,79,153]. Thus, the notion of executive dysfunctions as an underlying mechanism of neurodevelopmental difficulties in VP children is now well documented [15,82,131,154]. Executive deficit is central to the neurodevelopmental phenotype of preterm infants and their learning difficulties, both from a cognitive and a behavioral or social point of view [1,21,98,101,155].

The assessment and development support of EFs, as a whole, seem essential to support the development of prematurely born infants. Early and rapid assessments of EFs are possible, relying on observations of the child’s behavior, via parents or teachers (BRIEF). When premature children have reached school age or adolescence, they need to be able to have a complete evaluation of their neurodevelopment, which includes not only an evaluation of cognitive skills, praxis and executive functions by calibrated behavioral psychology tests, but also an evaluation of their behavior, their level of anxiety, their attentional capacities, their social interactions and their quality of life, via self- and hetero-questionnaires (parents, teachers, etc.).

The possibility of training and strengthening executive functions to optimize overall executive functioning and promote neurodevelopment has been explored in numerous studies, starting at preschool age, but also later, in childhood and adolescence. Very different modalities have been proposed, ranging from generalist interventions in a school environment, to much more specific and targeted interventions, such as computerized cognitive training, including the practice of mindfulness, sports or music [58,156,157]. The specific computerized training programs to support executive functioning have mainly focused on WM training with Cogmed^©^ software, or on a general approach to training all EFs with BrainGame Brian^©^ software. The results of these programs are currently disappointing in premature infants [29,131,158,159,160], even if they can improve one or more executive functions transitorily. Therefore, these should not be used as a standalone and can perhaps have a role in a comprehensive care package for EF support. Focusing on specific EF training was shown to be less effective in children aged from 4 to 12 than programs that integrated emotional and social components (Montessori-type school programs or “Tools of the Mind” in North America), including psychomotor components such as yoga or martial arts [156]. The authors conclude that the way to stimulate EFs is to take into account all the components of the child, including emotional, social and physical components [157]. This is certainly even truer for the specific population of VP infants for whom it is the entire neurodevelopment, in all its cognitive, behavioral and social components, that is impacted by premature birth [1,83]. A 2020 meta-analysis of cognitive training of young children to optimize their EFs which covered 30 studies published between 2009 and 2019 on children aged 3–6 years confirmed this trend [161]. The benefit of cognitive EF group training, such as the school-based type, is more effective than individual training. Motivation among peers and interaction with other children is a particularly significant support. In addition, in this same study, the non-computerized nature of the training brought a greater benefit: the use of card games, global or fine psychomotricity activities at this age seems more effective than the use of a computer. Finally, prior to all the programs offered to premature children, in terms of training, rehabilitation or remediation, there is the question of perinatal prevention strategies for neurodevelopmental disorders in the event of preterm birth. Numerous medical and technical advances in the perinatal care of these children have already been employed, such as inborn birth strategy, antenatal corticosteroid therapy and magnesium sulfate, optimal respiratory and nutritional support, and postnatal monitoring; other advances are certainly to come, such as the neuroprotective erythropoietin approach, for example [162,163], or caffeine as further neuroprotection [164]. Another essential avenue of prevention is the set of developmental care strategies applied in the neonatal period, such as skin-to-skin practice, the kangaroo method and the Newborn Individualized Developmental Care and Assessment (NIDCAP) program. Scientific data on the neurodevelopmental benefits of these developmental care techniques and programs bear witness to this [165,166,167]. Parents, considered as the main support for the neurodevelopment of the child in these programs, are put at the center of care. We can postulate that the parent–child bonds (attachment and bonding) created during this neonatal period [168,169,170] make therefore possible special attention and support to the neurobehavior of these very premature children throughout their early childhood.

## Figures and Tables

**Table 1:** Studies investigating the premature neurobehavioral phenotype.

Authors	Population	Method	Cluster Analysis	FSIQ	EFs	Behavior
Korzeniewski 2017 [95]	776 (<28 GA weeks) 10-year-old children/full term	Correlation of clinically significant high score on the Social Responsiveness Scale (SRS) in extremely premature and not meeting criteria for autism spectrum disorder (ASD)	Among children who had IQ ≥ 85, the prevalence of SRS total scores > 65 was 16% (*n* 103/628) and, among children who had IQ < 85, it was 27% (*n*: 40/148), higher than the 4% prevalence expected based on normative population data	After excluding 61 participants diagnosed with ASD, the authors grouped children by IQ < or ≥ 85 and then compared the prevalence of neurocognitive and other deficits between those who had SRS total and component scores ≥65 and their peers who had lower scores	Among children who had IQ ≥ 85, those who had high SRS scores more often than their peers had deficits in attention and executive functions and language and communication	High total SRS score > 65 were more often rated by their parents and teachers as having behavioral (e.g., attention-deficit hyperactivity disorder (ADHD)) and emotional (e.g., anxiety and depression) problems
Johnson 2018 [94]	1139 LMPT (from 32 to 36 weeks of GA)/1255 full term	Parent questionnaires were obtained to identify impaired cognitive and language development, behavioral problems, delayed social–emotional competence, autistic features and clinically significant eating	Two profiles were identified among the LMPT group (optimal, 67%; non optimal, 26%) (social, emotional and behavioral impairments). A third profile was identified (7%) that was similar to the phenotype previously identified in infants born very preterm.	Parent questionnaires	A smaller proportion of children born LMPT had impairments consistent with the “very preterm phenotype” which were likely to have arisen through a preterm pathway. Male sex, greater gestational age and pre-eclampsia were only associated with the preterm phenotype.	Two profiles of development among the term group, optimal (84%) and a profile of social, emotional and behavioral impairments termed “nonoptimal” (16%)
Heeren 2013 [57]	873 participants, age of 10 years, Elgan study <28 weeks of GA	Measures of FSIQ and EFs, subgroups of EP children with common neurocognitive functions, identified using latent profile analysis (LPA), nature and prevalence of impairment in EP children and examination of associations between cognitive function, GA and academic achievement	Four neurocognitive profiles in EP children, i.e., 34% of EP children classified as normal, 41% as low-normal, 17% as moderately impaired and 8% as severely impaired. Impaired children exhibited global impairment across the cognitive domain. Children in the low-normal group tended to have impaired inhibition relative to their reasoning and working memory skills.	Classification of neurocognitive functions using FSIQ and EFs were compared with a standard classification based on FSIQ Z-scores	Impaired children exhibited global impairment across cognitive domains, whereas children in the low-normal group tended to have impaired inhibition relative to their reasoning and working memory skills	Behavior: NA
Gire 2021 [1]	231 school-aged EPT children without severe sequelae	An algorithm distributed the study population according to four WISC-IV subtests, five NEPSY-2 subtests and two variables of figure of Rey. Behavior (SDQ), anxiety (Spielberg STAI-C) and QOL were evaluated between clusters (Kidscreen and VSPA)	Three neurobehavioral “phenotypes” were defined according to their severity, i.e., 1 = moderately, 2 = minor and 3 = unimpaired (with only emotional behavior and/or dysexcutive syndrome)	School-aged EPT children (7–10 years-old) without major disabilities, FSIQ > 70	Working memory and perceptual reasoning, as well as mental flexibility, were close to or below average	Emotional behavior was always troubled. QOL deterioration was determined by the severity of the profile. Self-esteem and school-work were the most impacted QOL areas.
Twilhaar 2021 [43]	1977 children born very preterm (<32 weeks of GA) in 2011 from the French-population-based EPIPAGE 2 cohort	Using latent profile analysis, subgroups of children were distinguished based on their functioning at 5.5 years. The relation between outcome profiles and neonatal and social/environmental factors was tested using multivariable multinomial logistic regression analysis.	Four subgroups with distinct outcome profiles were distinguished, i.e., no deficit in any domain (45%); motor and cognitive deficits without behavioral/psychosocial deficits (31%); primarily behavioral and psychosocial deficits (16%); and deficits in multiple domains (8%). Male sex (odds ratio (OR) = 2.1–2.7), bronchopulmonary dysplasia (OR = 2.1–2.8), low parental education level (OR = 1.8–2.1) and parental non-European immigrant status (OR = 2.3–3.0) were independently associated with higher odds for all suboptimal outcome profiles compared with the favorable outcome profile.	WPPSI-IV, MABC	NA Subgroups could be distinguished with distinct outcome profiles that varied in severity, type and combinations of deficits	SDQ
Boolk 2018 [96]	355 children, born at a GA of less than 27 weeks from April 2004 to March 2007 vs. 364 term-born controls at 6.5 years of age	Assessment of visual–motor integration, cognitive function, motor skills and vision. Visual–motor integration impairment was classified as <−1 standard deviation.	The mean (standard deviation) visual–motor integration score was 87 (±12) in preterm children compared to 98 (±11) in controls (*p* < 0.001). Visual–motor integration impairment was present in 55% of preterm infants and in 78% of children born at 22–23 weeks.	Male sex and postnatal steroids showed a weak association with poorer visual–motor performance, whereas low manual dexterity and cognitive function showed a stronger association.		
Ross 2016 [97]	117 children < 1250 g BW seen at 18 months post-term	Bayley Scales-III and Child Behavior Checklist 1 ½-5 (CBCL 1 ½-5), a behavioral problem questionnaire. Demographic and perinatal variables were obtained from medical records. Bayley Cognitive, Expressive Language and Receptive Language scores were used to cluster the subjects into developmental profiles	Four groups, i.e., consistently high, consistently average, average with delayed expressive language and consistently low. The study provides an informative means for identifying factors related to developmental outcomes.	Problems scores were significantly related to clusters.	Problems and attention deficit/hyperactivity (ADHD)	Socioeconomic status, bronchopulmonary dysplasia, Grades III–IV intraventricular hemorrhage and behavioral problems and attention deficit/hyperactivity (ADHD) problem scores were significantly related to clusters’ severity.

Studies illustrating the long-term outcome of prematurity: neurodevelopmental disorder evoking hypo connectivity with a neurobehavioral disorder comprising at least isolated behavioral disorders and/or association with a dysexecutive syndrome worsening with more neurological disorder comorbidities.
